# Identification of Taihang-chicken-specific genetic markers using genome-wide SNPs and machine learning: BREED-SPECIFIC SNPS OF TAIHANG CHICKEN

**DOI:** 10.1016/j.psj.2024.104585

**Published:** 2024-11-22

**Authors:** Fu Wei, Zhang Ran, Ding Hong, Wang Wenjun, Liu Huage, Zang Sumin, Zhou Rongyan

**Affiliations:** a Hebei Agricultural University, Baoding, Hebei province 071001, China; b Institute of Animal Science and Veterinary Medicine, Baoding, Hebei province 071000, China

**Keywords:** Taihang chicken, Specific SNPs, Machine learning, Breed indentification

## Abstract

Taihang is an indigenous breed in Hebei Province and has a long history of evolution. To uncover the genetic basis and protect the genetic resources, it is important to develop accurate markers to identify Taihang at the molecular level. In this study, a total of 137 individuals from Taihang and other 4 breeds were selected to construct a genome-wide SNP map. The population genetic structure analysis revealed clear differentiation among the five breeds. A total of 47 SNPs were identified for differentiating Taihang from other breeds based on the fixation index (FST), linkage disequilibrium (LD) pruning, and machine learning, further validated using principal component analysis (PCA) and genetic relationship matrix (GRM). The 47 SNPs were annotated to genes associated with production, growth and development, immunity, adaptation, and appearance. Overall, the combination of 47 SNPs enables precise identification of Taihang, which significantly contributes to the preservation of native genetic resources.

## Introdution

Local breeds have accumulated considerable genetic variation and polymorphism over several years of selection, and these local genetic resources can be used as excellent breeding materials to promote the rapid development of the seed industry (Luo et al., 2020). Leveraging the genetic diversity of local chicken breeds, new varieties can be developed to satisfy diverse market demands which fulfilling the market's need for a variety of chicken products (Rachman et al., 2024). Local breeds are excellent hybrid parents that can better adapt to specific environments. As a result, the preservation of local breeds has become increasingly important for maintaining sustainable animal husbandry (Gao et al., 2023). Accurate breed classification is required for the conservation and utilization of animal genetic resources (Liang, et al., 2024). Therefore, it is necessary to precisely identify livestock and poultry varieties and establish molecular identification cards for these varieties.

Genomics technology has been applied to construct specific labels and develop molecular markers to ensure the protection of genetic resources (Zeng et al., 2022). The single-nucleoside polymorphisms (SNPs) have become crucial molecular markers in genomic research due to their large quantity, widespread distribution, strong genetic stability, rapid detection speed, and high quality (Rasheed et al., 2017). Breed-specific SNPs, intimately linked to distinct breed characteristics, serve as crucial genetic markers for breeding programs (Lee et al., 2022). Precise breed identification is an essential step in genetic and genomic studies, and breed-specific SNPs could capture breed differences and hence could be used for breed assignment (Kumar et al., 2021). Several methods have been used for mining breed-informative SNPs in recent years, such as pairwise Wright's FST, informativeness for assignment, Delta, and MAF-LD (Zhao et al., 2023; Kumar et al., 2021). Furthermore, the integration of feature selection with machine learning algorithms has proven to be highly effective in identifying population-informative markers (Schiavo et al., 2024). The application of machine learning enables high accuracy in a short time, making it particularly suitable for breed identification (Liu et al., 2023). The accuracy rates are greater than 90 % with AdaBoost, Random Forest, and Decision Tree for identifying target chicken populations, and a limited number of marker sets can effectively classify a target group (Seo et al., 2021). Machine learning models have identified that using 23 or 48 SNPs can accurately determine breed with high accuracy of 95.2-98.4 % (Kumar et al., 2024).

Taihang is a renowned indigenous breed known for both its meat and egg production, and it represents an excellent genetic resource in Hebei Province (Zhang et al., 2024; Yue et al., 2020). Taihang, distinguished by its small body size, long tail feathers, hemp feathers, and green shanks, inhabits an environment entirely different from the other four breeds: Bian, Wenchang, Tibetan, and Lindian (Wu et al., 2022; Tian et al., 2023; Zhong et al., 2022; Liu et al., 2024). Currently, phenotypic identification serves as the primary method of identifying the Taihang, whereas precise identification with effective breed-specific genetic markers is absent. Therefore, population genetics analysis and machine learning methods were used to extract breed-specific genetic markers and to identify and differentiate the Taihang at the genome level, which is conducive to the development of a molecular identity card for Taihang.

## Materials and methods

### Ethics statement

All samples were collected and processed in strict accordance with protocols approved by the Institutional Animal Care and Use Committee of the Heibei Agricultural University Animal Care and Use Committee in China (Permit Number: HB/2019/03, Baoding City, Hebei Province, China).

### Animals and genomic requecincing data

**Fig. 1:**
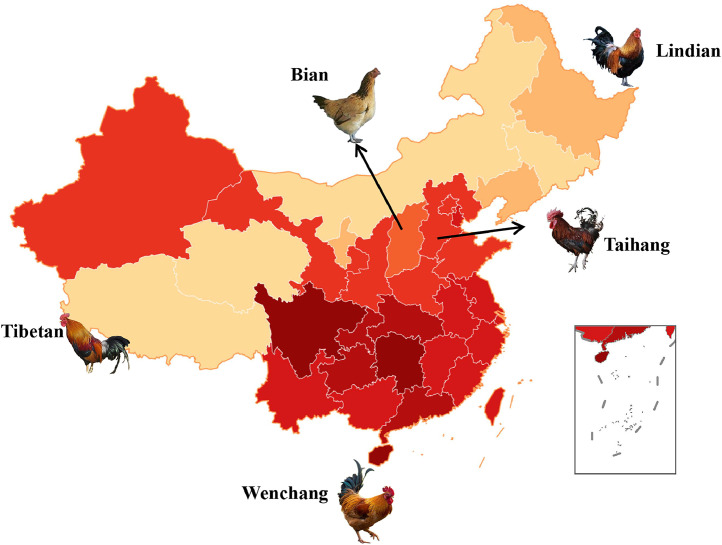
Geographic locations of five breeds.

We collected 40 blood samples from the Taihang genetic resources conservation population and extracted genomic DNA using a GenoPrep blood extraction kit. The quality and concentration of the extracted DNA were verified through 1 % agarose gel electrophoresis and by using the NanoDrop spectrophotometer 2000 (NanoDrop Inc., Wilmington, DE, USA). The 40 samples were resequenced on whole genome level by using the MGI-2000/MGI-T7 platform at the MolBreeding Company (Hebei province, China). Furthermore, we obtained whole-genome resequencing data from a dataset comprising 97 chickens from four distinct breeds: Tibetan (n = 30), Lindian (n = 26), Wenchang (n = 23), and Bian (n = 18), which were collected from various provinces (Fig. 1). The accession codes for the whole-genome resequencing datasets are PRJNA800119 and PRJNA698651. The original database contains sequence data for 137 chickens of 5 breeds.

### SNP calling and quality control

Quality control involved filtering out low-quality reads to obtain clean reads. Quality filtration involved removing (1) reads with ≧ 10 % unidentified nucleotides, (2) reads with > 50 % bases having Phred quality scores of ≦ 20, and (3) sequences of potential adapters and repeats. We then mapped the clean reads to the reference genome (GRCg6a, http://ftp.ensembl.org/pub/release-106/fasta/gallus_gallus/dna) using the BWA - mem algorithm (version 0.7.15) (Li, 2013). Duplicated reads were removed using Picard tools MarkDuplicates (https://github.com/broadinstitute/picard), and index files were created with SAMtools index (https://github.com/samtools/samtools). Subsequently, the 137 individual GVCFs were merged and called jointly to generate VCFs using the function CombineGVCFs and GenotypeGVCFs of GATK (version 4.0). Then, the function VariantFiltration of GATK (version 4.0) to filter SNPs with the following criteria parameters: Quality by depth (QD) < 2, Fisher strand (FS) > 60, Root mean square mapping quality (MQ) < 40, Strand odd ratio (SOR) > 3, Rank Sum Test for mapping qualities (MQRankSum) < − 12.5. SNPs that exhibited segregation distortions or sequencing errors were excluded, and quality control was performed on the SNP data utilizing the PLINK (version 1.90) (Chang et al., 2015). The following parameters are used for quality control: being biallelic, 100 % genotyping rate (not allowing any missing values), locating on autosomes, minor allele frequency ≧ 0.05, and Hardy-Weinberg equilibrium (*p*) < 1 × 10^−5^. These processes were completed in one step, ultimately we obtained 3,232,640 SNPs. These filtering criteria and quality control help to identify and exclude low-quality SNPs, thereby ensuring the reliability of subsequent analyses (Bayer et al., 2022).

### Population genetic structure analysis

PCA was conducted using PLINK the “-pca” option through a dimensionality reduction clustering approach in five breeds (Purcell et al., 2007). The neighbor-joining (**NJ**) tree was constructed based on pairwise distances using PHYLIP (version 3.697) (Baum, 1989), and visualized using the online tool iTOL (https://itol.embl.de). The ADMIXTURE (version 1.30) was used to infer the population structure (Alexander et al., 2009) and visualized in R (4.4.0).

### Selection of Taihang-breed-informative SNPs

Taihang-specific SNPs were extracted from the dataset using PLINK (version 1.90). The requirements for specific SNPs were as follows: (1) allele frequencies greater than 0.1, and (2) SNPs that are uniquely present in Taihang. FST is an important statistic to designate SNP as breed-specific. The value range of the FST statistic is between 0-1; the larger the number, the greater the separation degree of the population. The FST value of all SNP (-weir-fst-pop) between the Taihang and the four populations of Bian, Wenchang, Lindian, and Tibetan was calculated utilizing the VCFtools (version 0.1.15) (Danecek et al., 2011). The top 1 % SNPs were screened out according to FST values. Then, the SNPs were pruned with LD (LD-pruning) using PLINK (version 1.90) by the parameter (-indep-pairwise 50 10 0.1) to remove highly interlocking SNPs. One of the key steps in the breed identification procedure is: selection of the most breed-informative markers relevant to the breeds (Zhao et al,. 2024). By employing FST and LD-pruning, we removed a large number of non-contributing variables, thereby avoiding the subsequent machine learning to handle excessive information and reducing model confusion (Handelman et al,. 2018). Finally, a total of 260 SNPs rich in breed information were retained.

### Machine learning and ensemble learning for breed identification

The six machine learning models encompassing Support Vector Machine (**SVM**), K-Nearest Neighbor (**KNN**), Random Forest (**RF**), Decision Tree (**DT**), eXtreme Gradient Boosting (**XGBoost**), and Logistic Regression (**LR**) were utilized for screening SNPs. Prior to training the machine learning models, the genotype data was encoded as follows: reference homozygous genotype 0/0 was assigned a value of 0, heterozygous genotype 0/1 a value of 1, and alternative homozygous genotype 1/1 a value of 2. A binary classification problem, categorical features are simply binary and are either true (1) or false (0) (Greener et al,. 2022). All Taihang are designated as 1, while other chickens are designated as 0 in the label column of the model.

Libraries such as pandas, scikit-learn, matplotlib, and numpy are commonly utilized in Python (version 3.12.2) for the development of classification models. The methodology comprised the following steps: (1) partitioning the dataset into an 80 % training set and a 20 % testing set, (2) utilizing GridSearchCV to optimize hyperparameters for each model, (3) producing confusion matrices and Receiver Operating Characteristic (**ROC**) curves, and (4) assessing model performance using accuracy and the Matthews Correlation Coefficient (**MCC**) metrics (Alcantara et al., 2022).

$$\text{Accuracy}=\frac{\text{TP}+\text{TN}}{\text{TP}+\text{TN}+\text{FP}+\text{FN}}$$

$$\text{MCC}=\frac{\text{TP}\times \text{TN}-\text{FP}\times \text{FN}}{\sqrt{\left(\text{TP}+\text{FP}\right)\left(\text{TP}+\text{FN}\right)\left(\text{TN}+\text{FP}\right)\left(\text{TN}+\text{FN}\right)}}$$

where TP represents true positives, i.e. the number of individuals correctly predicted to be positive; FP represents false positives, i.e. the number of individuals incorrectly predicted to be positive that are actually negative; FN represents false negatives, i.e. the number of individuals incorrectly predicted to be negative that are actually positive; and TN represents true negatives, i.e. the number of individuals correctly predicted to be negative.

### Evaluating the discrimination power of SNPs

The SNPs were further validated using PCA, NJ tree, and GRM. Genetic relationships among individuals, commonly characterized by a GRM, have driven major advances in modern genetics (Fan et al., 2022). The GRM was computed using GCTA software (version 1.94.1) with the parameters '–make-grm' and '–make-grm-alg 0′ (Melinda et al., 2020). Three methods (mean_GRM, SD_GRM, and GRM_SVM) were devised based on GRM (Wilmot et al., 2023) using R packages (dplyr, caret, matrixStats, lattice, genefilter) for breed assignment (https://github.com/hwilmot675/Breed_assignment). Mean_GRM refers to the assignment based on the highest mean relatedness of an animal to the breeds. SD_GRM refers to the assignment based on the highest standard deviation (SD) of the relatedness of an animal to the breeds. GRM_SVM refers to the assignment based on a linear support vector machine (SVM) using the different values of mean and SD of the relatedness of the animal to be assigned as an input.

### Gene functional enrichment analysis

The specific SNPs were annotated to genes based on the gene annotation files utilizing the ANNOVAR script. These genes were then subjected to Gene Ontology (**GO**) term and Kyoto Encyclopedia of Genes and Genomes (**KEGG**) enrichment analyses using the KOBAS platform (http://bioinfo.org/kobas). Further examination focused on GO terms and KEGG pathways that exhibited significant enrichment (*P* < 0.05).

## Results

### Population structure of five breeds

**Fig. 2:**
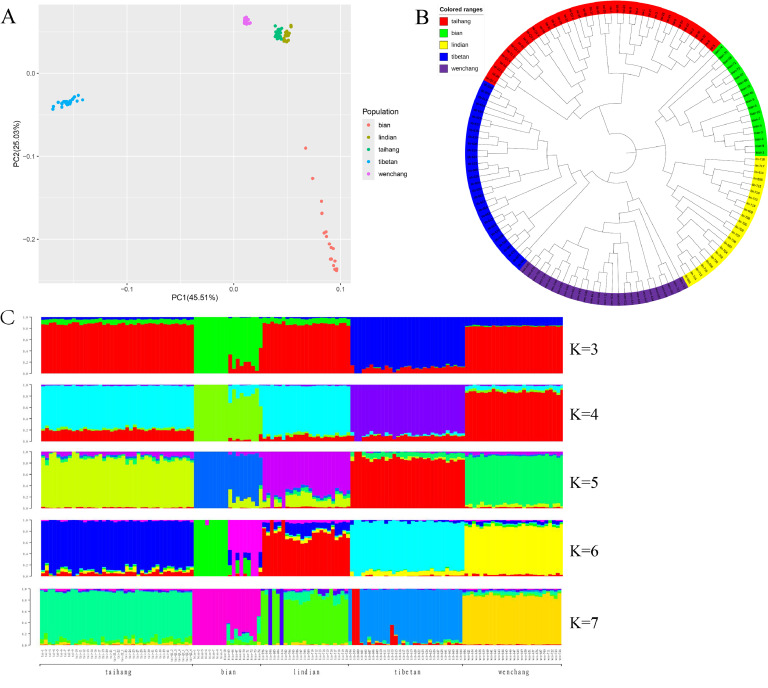
Population structure analyses of five breeds. (A) Principal component analysis of the 137 individuals. PCA1 and PCA2 explained 45.51 % and 25.03 % of the observed variance, respectively. (B) Neighbor-joining tree of 137 chickens. (C) Admixture analysis with K values running from 3 to 7.

After stringent quality filtering, 3,232,640 SNPs were used for population structure analysis. As resulted from the PCA-inferred breed distribution, there were compact localization and appropriate breed assignment of all the individuals (Fig. 2A). The NJ tree clearly separated into five clusters and also supported the PCA (Fig. 2B). The population genetic structure of five breeds was estimated by ADMIXTURE, with K values ranging from 3 to 7. We also found Taihang and Lindian clustered more closely with each other from K = 3 to K = 4 (Fig. 2C). A division between Taihang and Lindian was found at K = 5, with slight genetic admixture between these two breeds, indicating a close genetic relationship between the two breeds. This may be due to Lindian is related to the chicken breed brought by immigrants from Hebei, Shandong and other places in the Northern Famine (China National Commission of Animal Genetic Resources. 2010).

### Machine learning identifies 47 SNPs for Taihang breed identification

**Fig. 3:**
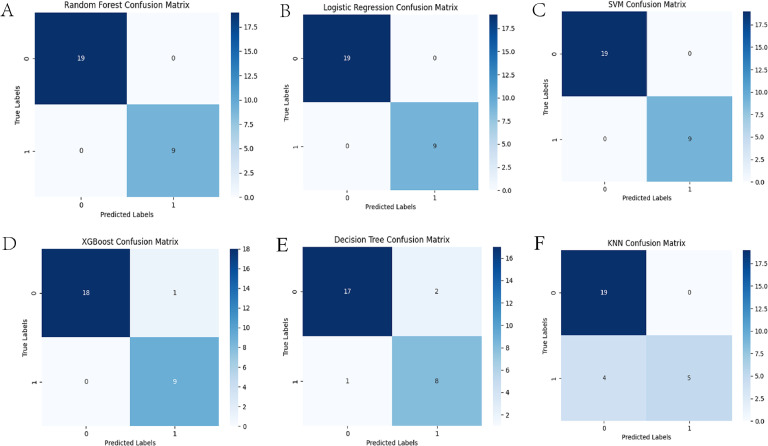
Results of confusion matrix for machine learning models. A, B, C, D, E, and F refer to the RF, LR, SVM, XGBoost, DT, KNN, respectively. RF, Random Forest; LR, Logistic Regression; SVM, Support Vector Machine; XGBoost, eXtreme Gradient Boosting; DT, Decision Tree; KNN, K-Nearest Neighbor.

**Fig. 4:**
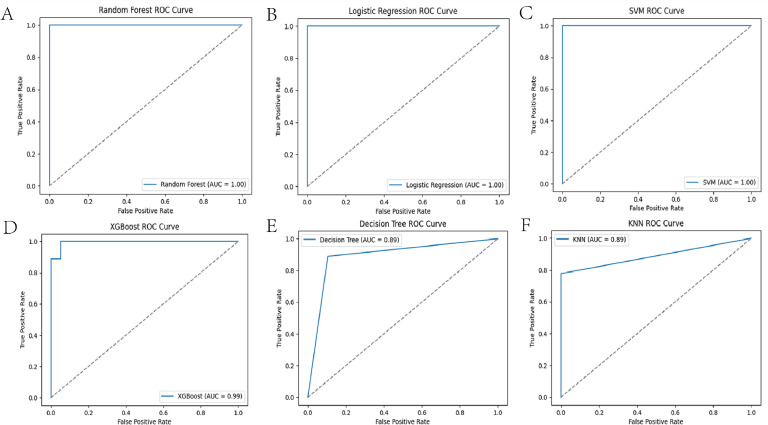
Results of ROC curve for machine learning models. A, B, C, D, E, and F refer to the RF, LR, SVM, XGBoost, DT, KNN, respectively. RF, Random Forest; LR, Logistic Regression; SVM, Support Vector Machine; XGBoost, eXtreme Gradient Boosting; DT, Decision Tree; KNN, K-Nearest Neighbor.

**Fig. 5:**
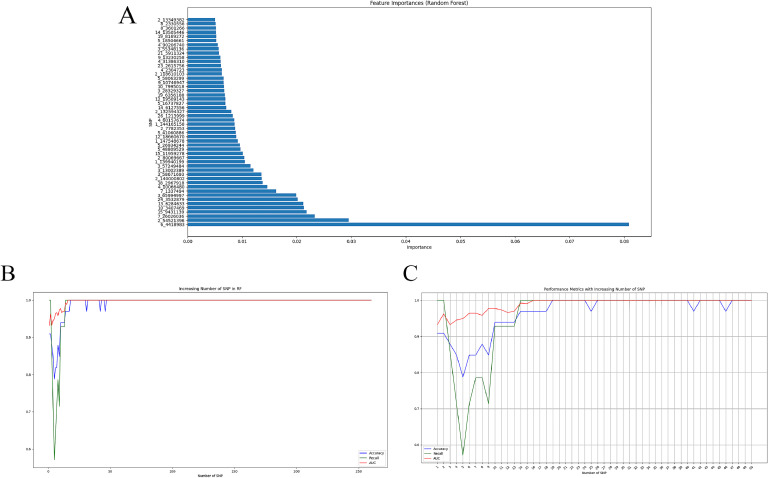
Results of random forest model analysis. (A) Top 50 SNPs with the highest scores in random forest analysis (the vertical coordinates are the positional coordinates of the SNP, 6-4418983 is meant to be the variant at locus 4418983 on chromosome 6). (B) Changes in Recall, Accuracy, and AUC of 260 SNPs. (C) Changes in Recall, Accuracy, and AUC of top 50 SNPs.

Following FST and LD-pruning analyses, 260 SNPs were selected for machine learning. The optimal hyperparameters of the models are shown in Supplementary Table S1. The confusion matrix provides a succinct summary of the model's performance, highlighting the number of correct and incorrect predictions for each class. The ROC curve is a valuable metric for assessing the model's discriminatory power, particularly when addressing unbalanced datasets. The results demonstrated the classification performance of the six models based on the 260 SNPs, all of which exhibited strong performance (Fig. 3, Fig. 4, and Table 1). The significance of 260 SNPs was assessed using the RF model (Fig. 5A), and we plotted the variations in three performance metrics of the test set (Fig. 5B). The results indicated that high accuracy, recall, and Area Under the Receiver Operating Characteristic Curve (**AUC**) are attained when the number of SNPs reached 47 (Fig. 5C).

**Table 1:** Classification accuracies and MCC values for the different machine learning models using the optimal combination of hyperparameters

model	MCC	Accuracy
RF	1.0000	1.0000
LR	1.0000	1.0000
SVM	1.0000	1.0000
XGBoost	0.9234	0.9600
DT	0.7638	0.8900
KNN	0.6774	0.8600

### Confirmation of the effectiveness of 47 SNPs in distinguishing Taihang

**Fig. 6:**
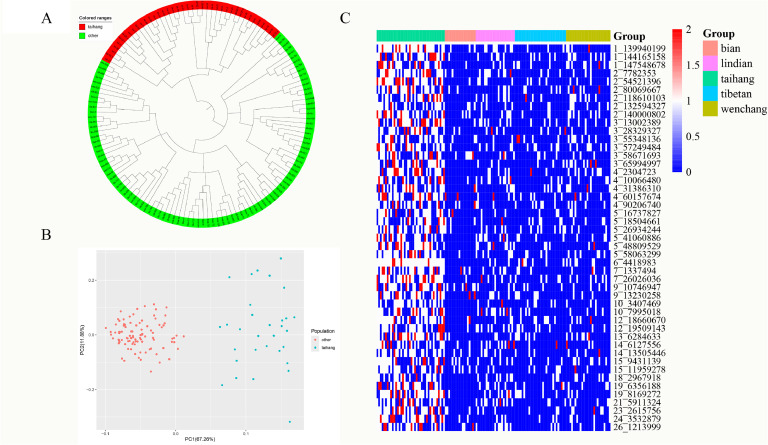
Verification of 47 specific SNPs. (A) Neighbor-joining tree between Taihang and other breeds. (B) Principal component analysis between Taihang and other breeds. PCA1 and PCA2 explained 67.26 % and 11.88 % of the observed variance, respectively. (C) The genotype heatmap of 47 specific SNPs in the five breeds. Blue, white, and red represent 0/0, 0/1, and 1/1 respectively.

PCA and NJ tree indicated that Taihang was accurately distinguished from other groups (Fig. 6). The GRM showed that all Taihang individuals were accurately classified as the Taihang population (Supplementary Table S2). The genotypic distribution of 47 SNPs significantly differs in Taihang and the other four populations, which further indicates the 47 SNPs could be used as molecular markers for identifying Taihang (Fig. 6).

### GO and KEGG analysis of 47 SNPs

**Fig. 7:**
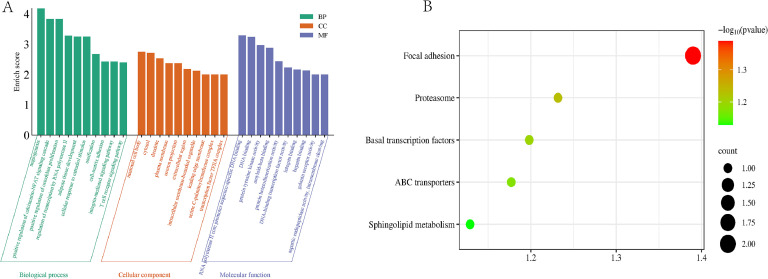
Functional enrichment analysis of 44 genes. (A) Top 10 GO terms of biological process, cellular component, and molecular function. (B) Top 5 enriched KEGG pathways. The size of circles for each pathway represents counts of associated genes. The color of the circles indicates the -log10(*P*-value).

To further analyze the functional implications of these 47 SNPs, we annotated them to their corresponding genes and biological pathways to explore the potential genetic mechanisms associated with various traits in chickens (Table S3, Fig. 7). Our gene annotation revealed that these candidate genes are linked to several significant traits in chickens, including appearance (*USP6*), production (*ACTBL2, WDR25, SORL1*), growth and development (*SLC10A7, EPHB2, TCF12*), adaptation (*RAMP3, CTGF, OPA1, PAQR9*), and immune responses (*FAM49B, FYN, H2AFZ, SPPL3, WDR90*) (Table 2). Gene ontology analysis indicated that angiogenesis (GO:0001525; *P* = 0.0000801) was the most significantly enriched term. Angiogenesis is crucial for essential life processes, such as the development of ovarian follicles (Chermuła et al., 2019) and bone tissue reconstruction (Huang et al., 2022). Additionally, KEGG enrichment analysis identified a single significant pathway, focal adhesion (gga04510; *P* = 0.044498056), which plays a vital role in key cellular activities, including adhesion, migration, proliferation, differentiation, and apoptosis.

**Table 2:** Candidate genes associated with important traits

Traits	Classification	Gene	Reference
production	meat quality	ACTBL2	Lamri et al., 2023.
	egg number	*WDR25*	Zhao et al., 2021.
	egg quality	*SORL1*	Zhang et al., 2011.
growth and development	bone	*SLC10A7; EPHB2*	Dubail et al., 2018; Zhang et al., 2020.
	muscle	*TCF12*	Li et al, 2022.
immunity	immune	*FAM49B; FYN; H2AFZ; SPPL3; WDR90*	Li et al., 2020; Yang et al., 2022; Zhang et al., 2023; Kemp et al., 2020; Valera-Pérez et al., 2019.
adaptation	heat tolerance	*RAMP3*	Guo et al., 2022.
	hypoxia tolerance	*CTGF*	Zhang et al., 2016; Zhang et al., 2022.
	cold tolerance	*OPA1;PAQR9*	Bosiacki et al., 2024; Liu et al., 2020.
appearance	feather	*USP6*	Davoodi et al., 2022.

## Disscussion

Germplasm resource conservation is a primary action for revitalizing the animal husbandry. The “Fourteenth 5-Year Plan” emphasizes organizing and carrying out accurate identification of livestock and poultry species (Liu et al., 2024). Breed identification is essential for protecting livestock pedigrees and for research (Cho et al,. 2022). Identifying chicken breeds at the genetic level could increase consumer trust in the market (Park et al., 2022). Taihang is an indigenous breed that can improve economic benefits and serve as an essential breeding material (Gao et al., 2024; Yang et al., 2023). This study provided the whole genome sequencing data of five breeds and analyzed the population structure, and constructed a molecular identity card of the Taihang, which can be used for the authenticity of the Taihang, and provide a solid scientific basis for the identification and protection.

The population structure of five breeds was analyzed based on whole genome resequencing data, revealing that Taihang has an independent genetic background. Then, we screened Taihang-specific SNPs using FST, LD-pruning, and machine learning. We employed six machine learning classification models, ultimately identifying 47 SNPs based on the random forest model. Three classification algorithms showed 100 % breed classification accuracy (Table 1). The may be attributed to the limited dataset, although we have mitigated the risk of overfitting through cross-validation and feature selection. Studies have demonstrated that achieving 100 % accuracy is feasible. Seven classification algorithms showed 100 % breed classification accuracy based on 96 SNPs when distinguishing the case and control groups (Seo et al., 2021). Furthermore, all eight machine learning algorithms successfully discriminated the Yeonsan Ogye population achieving accuracy, specificity, and precision up to 100 % (Cho et al,. 2022). A study has found that the guinea fowl required only four SNPs to attain 100 % through eight models, and the Cobb also necessitated only a minimal number of SNPs for the model to achieve 100 % (Zhi, 2023). Our findings are consistent with above studies showing that machine learning models require only a small number of features to achieve reliable identification performance in binary problems. To further substantiate the reliability of our results, we employed PCA, NJ tree and GRM to validate the classification efficacy, demonstrating that the SNPs are effective. The GRM has previously been applied for pedigree verification (Kaseja et al., 2022), estimating breeding values (Choi et al., 2017), and genomic best linear unbiased prediction (Pocrnic et al., 2019). Given the widespread application of GRM, it is feasible to extend its use to breed assignment. Overall, our results showed that Taihang could be distinguished by 47 SNPs.

The five breeds examined in this study originate from diverse environments: Tibetan, which are adapted to the low-oxygen conditions of the plateau; Wenchang, which thrive in tropical climates; Lindian, which are suited to cold environments; and Bian and Taihang, both adapted to temperate zones. The variations in genetic backgrounds and geographic locations have significantly influenced their adaptations to these distinct environments. Among the annotated genes, we identified four genes *RAMP3* (chr2:54521396), *CTGF* (chr3:57249484), *PAQR9* (chr9:10746947), *OPA1* (chr9:13230258) that may play a role in environmental adaptation. It is hypothesized that these genes are key factors contributing to the adaptation of these breeds to their respective environments. Receptor activity modifying protein 3 (***RAMP3***) plays a crucial role in glucose and energy homeostasis (Coester et al., 2020; Leuthardt et al., 2023). Previous studies have demonstrated that the expression of the *RAMP3* gene is elevated in response to high-fat and high-sucrose diets, indicating a potential association between *RAMP3* and both energy metabolism and thermogenesis (Pennington et al., 2020). Connective-tissue growth factor (***CTGF***) is a secreted protein involved in various cellular processes, including angiogenesis, skeletogenesis, and wound healing (Abreu et al., 2002), and is essential for normal lung development (Wang et al., 2019). A recent study highlighted the potential role of *CTGF* in adaptation to low-oxygen environments (Gomez et al., 2020). Oxygen supply is constantly maintained by the vascular network for a proper tissue oxygenation. Angiogenesis is one of the adaptive responses to hypoxia and is mainly regulated by the hypoxia-inducible factors (Monaci et al., 2024). Angiogenesis was enriched in our results and *CTGF* was enriched in this term (*P*=0.0000801). *CTGF* associated with cardiorespiratory development, indicating the crucial role of blood vessel development in the adaptation of Tibetan to hypoxic conditions (Zhang et al., 2016). *OPA1* is a key regulatory component of mitochondrial energetic adaptation, and these adaptations can significantly influence organismal fitness and survival in changing environments (Bennett et al., 2022). Previous studies have indicated that *OPA1* is essential for the cold-induced activation of thermogenic genes in brown adipose tissue, thereby mediating an adaptive response that enhances thermoregulation (Pereira et al., 2021). Progestin and adipoQ receptor family member 9 (***PAQR9***), a member of the PAQR family, plays a crucial role in metabolic processes (Li et al., 2023). A study has shown that the mRNA levels of *PAQR9* in brown adipose tissue experience significant upregulation in response to cold exposure, which is vital for activating thermogenesis in brown adipocytes (Liu et al., 2020). These candidate genes help to improve our understanding of adaptation in chickens.

## Conclusions

Taihang has a different genetic structure from the other four local chicken breeds. The 47 breed-specific SNPs can successfully separate Taihang from different populations. These results offer the potential for identifying and conserving the traceability of Taihang.

## Disclosures

The authors declare that they have no conflict of interest.

## Appendix Supplementary materials

Image, application 1
