# Supplementary material for: Identification of Taihang-chicken-specific genetic markers using genome-wide SNPs and machine learning: BREED-SPECIFIC SNPS OF TAIHANG CHICKEN
Source: Poult Sci. 2024 Nov 22;104(1):104585. doi: 10.1016/j.psj.2024.104585 (PMC11635733; doi:10.1016/j.psj.2024.104585)
Supplement: Supplementary file 1 [file mmc1.docx]

**Table S1.** The optimal hyperparameters of the models

| **model** | **optimal hyperparameters** |
| --- | --- |
| RF | max_depth: 5  n_estimators: 300 |
| LR | C: 0.01 |
| SVM | C: 1  gamma: 0.001 |
| xGBoost | learning_rate: 0.2  max_depth: 3  n_estimators: 100 |
| DT | max_depth: None |
| KNN | n_neighbors: 3 |

**Table S2**. Mean relatedness and predicted results for five breeds based on genetic relationship matrix (GRM)

| **Bian** | **Lindian** | **Tibetan** | **Taihang** | **Wenchang** | **True** | **Predicted** |
| --- | --- | --- | --- | --- | --- | --- |
| -0.4481 | -0.2551 | -0.4118 | 0.5486 | -0.3294 | Taihang | Taihang |
| -0.3932 | -0.2067 | -0.3183 | 0.4206 | -0.1987 | Taihang | Taihang |
| -0.3981 | -0.2161 | -0.3446 | 0.4827 | -0.3114 | Taihang | Taihang |
| -0.3803 | -0.2000 | -0.3602 | 0.4675 | -0.2733 | Taihang | Taihang |
| -0.3696 | -0.2326 | -0.3898 | 0.5084 | -0.3230 | Taihang | Taihang |
| -0.3505 | -0.3005 | -0.3793 | 0.5160 | -0.3264 | Taihang | Taihang |
| -0.4422 | -0.2081 | -0.3465 | 0.4861 | -0.2903 | Taihang | Taihang |
| -0.2166 | -0.1183 | -0.2138 | 0.2681 | -0.1404 | Taihang | Taihang |
| -0.4123 | -0.2351 | -0.3788 | 0.5123 | -0.3144 | Taihang | Taihang |
| -0.2944 | -0.2717 | -0.2573 | 0.3918 | -0.1870 | Taihang | Taihang |
| -0.3952 | -0.2869 | -0.3685 | 0.5065 | -0.2712 | Taihang | Taihang |
| -0.4059 | -0.2386 | -0.3524 | 0.4958 | -0.2954 | Taihang | Taihang |
| -0.3740 | -0.1769 | -0.2982 | 0.4246 | -0.2526 | Taihang | Taihang |
| -0.3046 | -0.0933 | -0.2235 | 0.3221 | -0.2161 | Taihang | Taihang |
| -0.4076 | -0.1987 | -0.3202 | 0.4501 | -0.2498 | Taihang | Taihang |
| -0.3814 | -0.1338 | -0.2732 | 0.3560 | -0.1769 | Taihang | Taihang |
| -0.1948 | -0.0964 | -0.1385 | 0.2175 | -0.1479 | Taihang | Taihang |
| -0.2180 | -0.1173 | -0.1794 | 0.2589 | -0.1446 | Taihang | Taihang |
| -0.2999 | -0.2268 | -0.2332 | 0.3591 | -0.1858 | Taihang | Taihang |
| -0.3303 | -0.1612 | -0.2736 | 0.3784 | -0.2050 | Taihang | Taihang |
| -0.1572 | -0.0496 | -0.1529 | 0.1854 | -0.0948 | Taihang | Taihang |
| -0.3945 | -0.2326 | -0.3544 | 0.4859 | -0.2893 | Taihang | Taihang |
| -0.4269 | -0.2495 | -0.3711 | 0.5120 | -0.3014 | Taihang | Taihang |
| -0.4086 | -0.1991 | -0.3558 | 0.4731 | -0.2645 | Taihang | Taihang |
| -0.3013 | -0.1183 | -0.2257 | 0.3162 | -0.1988 | Taihang | Taihang |
| -0.2024 | -0.0865 | -0.1522 | 0.2219 | -0.1284 | Taihang | Taihang |
| -0.2556 | -0.2038 | -0.2446 | 0.3587 | -0.2150 | Taihang | Taihang |
| -0.4075 | -0.2446 | -0.3756 | 0.5047 | -0.3014 | Taihang | Taihang |
| -0.1836 | -0.0799 | -0.1970 | 0.2462 | -0.1485 | Taihang | Taihang |
| -0.1870 | -0.1283 | -0.1724 | 0.2608 | -0.1672 | Taihang | Taihang |
| -0.3769 | -0.2174 | -0.3170 | 0.4476 | -0.2813 | Taihang | Taihang |
| -0.1489 | -0.0994 | -0.1400 | 0.1868 | -0.0760 | Taihang | Taihang |
| -0.3605 | -0.1838 | -0.3029 | 0.4322 | -0.2678 | Taihang | Taihang |
| -0.1831 | -0.0740 | -0.1486 | 0.2151 | -0.1497 | Taihang | Taihang |
| -0.2798 | -0.1643 | -0.2405 | 0.3292 | -0.1605 | Taihang | Taihang |
| -0.3204 | -0.1880 | -0.2494 | 0.3558 | -0.1865 | Taihang | Taihang |
| -0.3734 | -0.2103 | -0.3333 | 0.4590 | -0.2620 | Taihang | Taihang |
| -0.2585 | -0.2149 | -0.2518 | 0.3647 | -0.2020 | Taihang | Taihang |
| -0.5034 | -0.2825 | -0.4793 | 0.6417 | -0.4065 | Taihang | Taihang |
| -0.3618 | -0.1836 | -0.2630 | 0.3966 | -0.2416 | Taihang | Taihang |
| 0.2929 | 0.1231 | 0.2298 | -0.2890 | 0.1697 | other | other |
| 0.3845 | 0.1831 | 0.3047 | -0.4039 | 0.2488 | other | other |
| 0.3024 | 0.1467 | 0.2601 | -0.3277 | 0.2064 | other | other |
| 0.3299 | 0.1698 | 0.2859 | -0.3599 | 0.2134 | other | other |
| 0.3355 | 0.1554 | 0.2530 | -0.3332 | 0.2018 | other | other |
| 0.3623 | 0.1667 | 0.2805 | -0.3625 | 0.2109 | other | other |
| 0.3770 | 0.1767 | 0.2889 | -0.3824 | 0.2334 | other | other |
| 0.3411 | 0.1780 | 0.2725 | -0.3594 | 0.2177 | other | other |
| 0.3127 | 0.1338 | 0.2423 | -0.3128 | 0.1907 | other | other |
| 0.3708 | 0.1836 | 0.2806 | -0.3806 | 0.2334 | other | other |
| 0.3659 | 0.1717 | 0.2735 | -0.3655 | 0.2153 | other | other |
| 0.3758 | 0.1995 | 0.3018 | -0.4028 | 0.2433 | other | other |
| 0.3469 | 0.1356 | 0.2295 | -0.3194 | 0.1954 | other | other |
| 0.2990 | 0.1401 | 0.2190 | -0.2926 | 0.1737 | other | other |
| 0.2269 | 0.0371 | 0.1005 | -0.1338 | 0.0601 | other | other |
| 0.3985 | 0.2091 | 0.3246 | -0.4275 | 0.2557 | other | other |
| 0.4034 | 0.1716 | 0.2492 | -0.3460 | 0.1872 | other | other |
| 0.2570 | 0.0500 | 0.1543 | -0.1863 | 0.1061 | other | other |
| 0.1442 | 0.1548 | 0.1441 | -0.1976 | 0.1127 | other | other |
| 0.1336 | 0.1094 | 0.1103 | -0.1546 | 0.1011 | other | other |
| 0.2429 | 0.1845 | 0.2360 | -0.3008 | 0.1711 | other | other |
| 0.0548 | 0.1593 | 0.0573 | -0.0952 | 0.0363 | other | other |
| 0.3137 | 0.1925 | 0.2668 | -0.3630 | 0.2348 | other | other |
| 0.2108 | 0.1845 | 0.1890 | -0.2560 | 0.1377 | other | other |
| 0.0583 | 0.1520 | 0.0760 | -0.1149 | 0.0540 | other | other |
| 0.1345 | 0.1516 | 0.1526 | -0.1902 | 0.1005 | other | other |
| 0.2077 | 0.1773 | 0.1966 | -0.2569 | 0.1399 | other | other |
| 0.2258 | 0.1666 | 0.2052 | -0.2671 | 0.1603 | other | other |
| 0.1258 | 0.1246 | 0.1033 | -0.1421 | 0.0770 | other | other |
| 0.1516 | 0.0847 | 0.1240 | -0.1484 | 0.0771 | other | other |
| 0.2248 | 0.1582 | 0.2092 | -0.2786 | 0.1778 | other | other |
| 0.1488 | 0.1381 | 0.1485 | -0.1983 | 0.1267 | other | other |
| 0.1327 | 0.1731 | 0.0860 | -0.1655 | 0.0975 | other | other |
| 0.1799 | 0.1377 | 0.1754 | -0.2037 | 0.0994 | other | other |
| 0.0882 | 0.1569 | 0.0682 | -0.1182 | 0.0541 | other | other |
| 0.0873 | 0.1019 | 0.0465 | -0.0845 | 0.0355 | other | other |
| 0.1230 | 0.2071 | 0.1168 | -0.1907 | 0.1011 | other | other |
| 0.0909 | 0.1242 | 0.0871 | -0.1163 | 0.0570 | other | other |
| -0.0019 | 0.1305 | 0.0084 | -0.0413 | 0.0096 | other | other |
| 0.1453 | 0.1260 | 0.1394 | -0.1850 | 0.1242 | other | other |
| 0.2677 | 0.1788 | 0.1962 | -0.2783 | 0.1606 | other | other |
| 0.2553 | 0.1288 | 0.2483 | -0.2882 | 0.1672 | other | other |
| 0.2042 | 0.1099 | 0.2241 | -0.2481 | 0.1542 | other | other |
| 0.1790 | 0.0882 | 0.2092 | -0.2174 | 0.1361 | other | other |
| 0.2942 | 0.1824 | 0.2842 | -0.3453 | 0.1921 | other | other |
| 0.2948 | 0.1577 | 0.2902 | -0.3438 | 0.2093 | other | other |
| 0.2727 | 0.1344 | 0.2651 | -0.3127 | 0.1880 | other | other |
| 0.2185 | 0.1515 | 0.2398 | -0.2933 | 0.2004 | other | other |
| 0.2776 | 0.1480 | 0.2506 | -0.3089 | 0.1859 | other | other |
| 0.2707 | 0.1489 | 0.2356 | -0.2986 | 0.1780 | other | other |
| 0.1666 | 0.0701 | 0.1562 | -0.1868 | 0.1400 | other | other |
| 0.2925 | 0.1411 | 0.2851 | -0.3406 | 0.2172 | other | other |
| 0.2302 | 0.0868 | 0.2200 | -0.2456 | 0.1471 | other | other |
| 0.2522 | 0.1437 | 0.2576 | -0.2945 | 0.1656 | other | other |
| 0.3308 | 0.1636 | 0.2903 | -0.3584 | 0.2137 | other | other |
| 0.2703 | 0.1219 | 0.2355 | -0.2805 | 0.1618 | other | other |
| 0.2340 | 0.1350 | 0.2346 | -0.2725 | 0.1595 | other | other |
| 0.3299 | 0.1726 | 0.2980 | -0.3626 | 0.2085 | other | other |
| 0.3031 | 0.1672 | 0.2669 | -0.3365 | 0.1980 | other | other |
| 0.2110 | 0.1147 | 0.2210 | -0.2532 | 0.1576 | other | other |
| 0.2273 | 0.1329 | 0.1908 | -0.2453 | 0.1529 | other | other |
| 0.2101 | 0.1507 | 0.2037 | -0.2634 | 0.1731 | other | other |
| 0.2662 | 0.1617 | 0.2540 | -0.3157 | 0.1976 | other | other |
| 0.2911 | 0.1668 | 0.2798 | -0.3511 | 0.2302 | other | other |
| 0.2695 | 0.1271 | 0.2550 | -0.2940 | 0.1709 | other | other |
| 0.1681 | 0.0913 | 0.1501 | -0.1736 | 0.0950 | other | other |
| 0.2029 | 0.1037 | 0.1869 | -0.2284 | 0.1464 | other | other |
| 0.2446 | 0.1198 | 0.2367 | -0.2699 | 0.1503 | other | other |
| 0.2754 | 0.1432 | 0.2502 | -0.2988 | 0.1684 | other | other |
| 0.2342 | 0.1469 | 0.2556 | -0.3007 | 0.1842 | other | other |
| 0.3076 | 0.1888 | 0.2973 | -0.3591 | 0.2039 | other | other |
| 0.1850 | 0.1094 | 0.1786 | -0.2324 | 0.1833 | other | other |
| 0.0988 | 0.0422 | 0.1042 | -0.1323 | 0.1281 | other | other |
| 0.0282 | 0.0541 | 0.0501 | -0.0788 | 0.0951 | other | other |
| 0.0715 | -0.0158 | 0.0546 | -0.0621 | 0.0827 | other | other |
| 0.1736 | 0.0328 | 0.1020 | -0.1449 | 0.1296 | other | other |
| 0.0795 | 0.0108 | 0.0763 | -0.0930 | 0.1176 | other | other |
| 0.1412 | 0.0777 | 0.1582 | -0.1831 | 0.1349 | other | other |
| 0.2670 | 0.1572 | 0.2300 | -0.3085 | 0.2104 | other | other |
| 0.2811 | 0.1417 | 0.2260 | -0.3001 | 0.2003 | other | other |
| 0.3105 | 0.1578 | 0.2889 | -0.3538 | 0.2291 | other | other |
| 0.2713 | 0.1412 | 0.2488 | -0.3282 | 0.2480 | other | other |
| 0.2093 | 0.1142 | 0.1844 | -0.2437 | 0.1779 | other | other |
| 0.2162 | 0.1083 | 0.1931 | -0.2538 | 0.1976 | other | other |
| 0.1936 | 0.1266 | 0.1809 | -0.2406 | 0.1845 | other | other |
| 0.0659 | 0.0065 | 0.0596 | -0.0824 | 0.1185 | other | other |
| 0.2142 | 0.1270 | 0.1784 | -0.2367 | 0.1607 | other | other |
| 0.2290 | 0.1138 | 0.1655 | -0.2303 | 0.1088 | other | other |
| 0.1497 | 0.1049 | 0.1645 | -0.2202 | 0.1946 | other | other |
| 0.3141 | 0.1996 | 0.2845 | -0.3810 | 0.2546 | other | other |
| 0.1561 | 0.0531 | 0.0958 | -0.1624 | 0.1838 | other | other |
| 0.2863 | 0.1453 | 0.2517 | -0.3304 | 0.2325 | other | other |
| 0.2851 | 0.1741 | 0.2497 | -0.3379 | 0.2247 | other | other |
| 0.2226 | 0.1223 | 0.1915 | -0.2506 | 0.1723 | other | other |
| 0.2089 | 0.1316 | 0.1940 | -0.2451 | 0.1580 | other | other |
| 0.2758 | 0.1875 | 0.2510 | -0.3334 | 0.2149 | other | other |
| 0.2118 | 0.1410 | 0.1905 | -0.2608 | 0.1924 | other | other |

**Table S3.** Details information of the 47 SNPs in this study

| **Chr** | **Position** | **SNPs** | **Gene** | **Type of variant** |
| --- | --- | --- | --- | --- |
| 1 | 139940199 | G/A | *MYO16、*ENSGALG00000049379 | intergenic variant |
| 1 | 144165158 | T/C | *METTL21EP、*ENSGALG00000048920 | intergenic variant |
| 1 | 147548678 | A/G | *ABCC4* | intronic variant |
| 2 | 7782353 | G/A | *PAXIP1* | intronic variant |
| 2 | 54521396 | G/A | *RAMP3* | intronic variant |
| 2 | 80069667 | A/G | ENSGALG00000048301、ENSGALG00000013079 | intergenic variant |
| 2 | 118610103 | C/G | *CRISPLD1*、*HNF4G* | intergenic variant |
| 2 | 132594327 | T/C | *SYBU* | intronic variant |
| 2 | 140000802 | T/C | *FAM49B、*ENSGALG00000033453 | intergenic variant |
| 3 | 13002389 | G/A | *SPTLC3、*ENSGALG00000050642 | intergenic variant |
| 3 | 28329327 | A/G | ENSGALG00000054135、ENSGALG00000010050 | intergenic variant |
| 3 | 55348136 | T/A | *BCLAF1* | intronic variant |
| 3 | 57249484 | T/C | *CTGF* | downstream variant |
| 3 | 58671693 | A/T | *PTPRK* | intronic variant |
| 3 | 65994997 | T/A | *FYN* | intronic variant |
| 4 | 2304723 | G/A | *ZMYM3* | intronic variant |
| 4 | 10066480 | G/A | ENSGALG00000052590 | ncRNA_intronic variant |
| 4 | 31386310 | G/A | *SLC10A7* | intronic variant |
| 4 | 60157674 | C/A | *H2AFZ* | upstream variant |
| 4 | 90206740 | G/A | ENSGALG00000048407 | ncRNA_intronic variant |
| 5 | 16737827 | C/T | *PPP6R3*、*GAL* | intergenic variant |
| 5 | 18504661 | G/A | *ACTBL2、*ENSGALG00000050964 | intergenic variant |
| 5 | 26934244 | A/G | *RGS6* | intronic variant |
| 5 | 41060886 | C/T | *GTF2A1*、*STON2* | intergenic variant |
| 5 | 48809529 | A/T | *WDR25* | intronic variant |
| 5 | 58063299 | C/T | ENSGALG00000012361 | intronic variant |
| 6 | 4418983 | A/C | ENSGALG00000047881、ENSGALG00000050807 | intergenic variant |
| 7 | 1337494 | T/C | *FAM171B*、*ITGAV* | intergenic variant |
| 7 | 26026036 | G/A | *TFCP2L1、*ENSGALG00000053618 | intergenic variant |
| 9 | 10746947 | A/G | *PCOLCE2、PAQR9* | intergenic variant |
| 9 | 13230258 | T/C | *OPA1* | intronic variant |
| 10 | 3407469 | C/G | *HMG20A、*ENSGALG00000049128 | intergenic variant |
| 10 | 7995018 | G/A | *TCF12* | intronic variant |
| 12 | 18660670 | C/T | ENSGALG00000053492 | ncRNA_intronic variant |
| 12 | 19509143 | C/T | *LMCD1、*ENSGALG00000054845 | intergenic variant |
| 13 | 6284633 | T/A | *TENM2、*ENSGALG00000047213 | intergenic variant |
| 14 | 6127556 | G/A | *SOX8、*ENSGALG00000051741 | intergenic variant |
| 14 | 13505446 | G/A | *WDR90* | intronic variant |
| 15 | 9431139 | A/G | *SPPL3* | intronic variant |
| 15 | 11959278 | A/G | *MED13L* | intronic variant |
| 18 | 2967918 | G/T | ENSGALG00000050670 | ncRNA_intronic variant |
| 19 | 6356188 | C/T | *SLC6A4* | intronic variant |
| 19 | 8169272 | T/C | *USP6* | upstream variant |
| 21 | 5911324 | G/A | *EPHB2、*ENSGALG00000026658 | intergenic variant |
| 23 | 2615756 | A/G | *RUNX3* | intronic variant |
| 24 | 3532879 | T/G | *SORL1* | intronic variant |
| 26 | 1213999 | T/C | *PSMA5* | intronic variant |
